# Alcohol-Related Liver Disease: Basic Mechanisms and Clinical Perspectives

**DOI:** 10.3390/ijms22105170

**Published:** 2021-05-13

**Authors:** Szu-Yi Liu, I-Ting Tsai, Yin-Chou Hsu

**Affiliations:** 1 Department of Emergency Medicine, E-Da Hospital, I-Shou University, Kaohsiung 82445, Taiwan; sizi0011@yahoo.com.tw (S.-Y.L.); tsaiting@msn.com (I.-T.T.); 2 School of Medicine for International Student, I-Shou University, Kaohsiung 82445, Taiwan; 3 School of Chinese Medicine for Post Baccalaureate, I-Shou University, Kaohsiung 82445, Taiwan

**Keywords:** alcoholic liver disease, alcohol use disorder, pathogenesis, epigenetics, diagnose, biomarker, micro RNA, therapy, relapse

## Abstract

Alcohol-related liver disease (ALD) refers to the liver damage occurring due to excessive alcohol consumption and involves a broad spectrum of diseases that includes liver steatosis, steatohepatitis, hepatitis, cirrhosis, and hepatocellular carcinoma (HCC). The progression of ALD is mainly associated with the amount and duration of alcohol usage; however, it is also influenced by genetic, epigenetic, and environmental factors. The definite diagnosis of ALD is based on a liver biopsy, although several non-invasive diagnostic tools and serum biomarkers have emerging roles in the early detection of ALD. While alcohol abstinence and nutritional support remain the cornerstone of ALD treatment, growing evidence has revealed that the therapeutic agents that target oxidative stress or gut-liver axis, inflammatory response inhibition, and liver regeneration enhancement also play a role in ALD management. Furthermore, microRNAs modulation and mesenchymal stem cell-based therapy have emerging potential as ALD therapeutic options. This review summarizes the updated understanding of the pathophysiology, diagnosis, and novel therapeutic approaches for ALD.

## 1. Introduction

Chronic alcohol consumption is one of the most common causes of morbidity and mortality worldwide and has an impact on more than 200 disease and injury outcomes [1,2]. According to the World Health Organization (WHO), 2.3 billion people are current drinkers, and approximately 1 billion people are classified as heavy episodic drinkers [3]. The alcohol-attributable fraction is the highest for liver cirrhosis, followed by road injury and other digestive diseases [3]. Alcohol is a well-recognized carcinogen in several types of cancers and has an established relationship with other liver-specific disease progression, such as chronic viral hepatitis and hepatocellular carcinoma (HCC) [4,5,6,7].

The liver is the primary organ responsible for ethanol metabolism, suffering from greater tissue injury through oxidative stress, acetaldehyde, and lipopolysaccharide (LPS) accumulation after excessive alcohol consumption [8,9]. Alcohol-related liver disease (ALD) refers to a broad spectrum of diseases, including asymptomatic early ALD (fatty liver or steatosis), steatohepatitis, advanced ALD (alcoholic hepatitis, cirrhosis), and HCC attributable to alcohol consumption [10,11].

Although there is a clear correlation between the amount and duration of alcohol intake and ALD progression, it is postulated that other co-factors (e.g., genetic, epigenetic, and environment factors) also play a role in ALD development, as only 10–20% of individuals with chronic alcohol use will progress to advanced ALD [12].

The prompt diagnosis of early ALD and complete alcohol abstinence is crucial in the ALD treatment strategy, as irreversible liver damage and hepatic decompensation have not occurred at this stage [12,13]. Nevertheless, no reliable symptoms, signs, or biochemical tests could aid in an early ALD diagnosis; additionally, the histological features of ALD may be similar to those of nonalcoholic fatty liver disease (NAFLD) [12,14]. Several non-invasive tests, including serum biomarkers and elastography, have been proposed for the detection and staging of ALD in a more accurate manner [2]. Moreover, a number of therapeutic agents that target oxidative stress [15], microRNA [16], gut microbiota modulation [17], and mesenchymal stem cell-based therapy [18] have been investigated in ALD prevention and treatment plans. In this review article, we summarize the basic mechanisms regarding the pathophysiology, diagnosis, and clinical management strategies, including molecule-based therapeutic agents in ALD.

## 2. Pathophysiology

### 2.1. Ethanol Metabolism

Alcohol is absorbed through the gastrointestinal tract into the blood circulation and is mainly metabolized by hepatocytes in the liver [19]. There are three main enzymatic metabolic pathways responsible for alcohol metabolism within the hepatocytes [20].

The first and the main pathway is hepatocyte cytoplasmic alcohol dehydrogenase (ADH), which uses nicotinamide adenine dinucleotide (NAD^+^) as a co-factor and oxidizes ethanol to acetaldehyde, which is highly toxic and causes DNA synthesis impairment [8]. The second pathway is the microsomal ethanol-oxidizing system (MEOS) in the smooth endoplasmic reticulum, which requires the cytochrome P450 2E1 (CYP2E1) enzyme to oxidize ethanol to acetaldehyde, generating reactive oxygen species (ROS), and triggering oxidative stress and inflammation [8,21]. It is noteworthy that CYP2E1 only catalyzes approximately 10% of ethanol into acetaldehyde under normal physiological conditions; however, it becomes more prominent in chronic alcohol consumption due to enhanced CYP2E1 expression [8,9]. The third and more minor pathway is via the heme-containing catalase in the peroxisomes, which can also oxidize ethanol to acetaldehyde [22]. The enzyme aldehyde dehydrogenase (ALDH) is located in the hepatocyte mitochondria and further oxidizes acetaldehyde to acetate, which is released into the circulation system and is further oxidized to carbon dioxide in various extrahepatic tissues [9].

### 2.2. Mechanisms in ALD Development

Although complex and not fully understood, the underlying molecular mechanisms responsible for ALD development include direct ethanol hepatotoxicity and lipid peroxidation, oxidative stress and ROS production, immune response activation and cytokine accumulation, and hepatic metabolism disorder [19,23,24]. 

#### 2.2.1. Direct Ethanol Hepatotoxicity and Lipid Peroxidation

As mentioned earlier, chronic alcohol use upregulates CYP2E1 production, which leads to increased acetaldehyde concentration, diminished ALDH activity, and reduced acetaldehyde oxidation, resulting in acetaldehyde accumulation, which directly damages the mitochondria and microtubules of hepatocytes in the liver [19,20]. Furthermore, ethanol and acetaldehyde downregulate adiponectin, signal transducer and activator of transcription 3 (STAT3), and zinc levels, further inhibiting 5′-AMP-activated protein kinase (AMPK), peroxisome proliferator activated receptor α (PPARα), and its target gene activity, resulting in phospholipid peroxidation and lipid free radical production, and enhanced early growth response protein 1 (Egr-1) and adiponectin and acetyl-CoA carboxylase (ACC) expression, all of which cause fatty acid accumulation in the liver [25,26]. Recent studies also provided evidence that the lipogenesis process by lipolysis and free fatty acid flux to the liver from the small intestine further decreased the adipose tissue mass in an animal model with chronic alcohol consumption [27,28].

#### 2.2.2. ROS Production and Oxidative Stress

CYP2E1-mediated or ethanol-induced inflammatory oxidative stress causes ROS generation (e.g., superoxide, hydroxyl radicals), which can bind to proteins and result in structural or functional alterations [15,26]. ROS can also bind directly to DNA and generate highly carcinogenic exocyclic ε-DNA adducts, which exhibit high mutagenic potential in numerous types of base pair substitutions and genetic damage in organisms, and has been analyzed as a representative lipid peroxidation-derived DNA damage marker in several studies [29,30]. In addition, acetaldehyde-mediated glutathione decreases and dysregulates the expression of antioxidant genes, including nuclear factor erythroid 2-related factor 2 (Nrf-2) and thioredoxin, leading to decreased antioxidant and detoxification enzyme production, and low activity of the antioxidant defense system [26,31].

#### 2.2.3. Cytokines Activation and Advanced Fibrogenesis

Chronic alcohol consumption is a known factor of intestinal endotoxin accumulation and intestinal wall permeability increasing, facilitating the translocation of endotoxins from the intestines to the liver in the form of LPS, which are toxic to hepatocytes [9]. LPS can bind to different toll-like receptors (TLRs) and activate the synthesis and release of cytokines and inflammatory factors, such as tumor necrosis factor α (TNF-α), interlukin-1 (IL-1), interlukin-6 (IL-6), and platelet-derived growth factor (PDGF), further stimulating neutrophil and macrophage accumulation, and finally causing hepatic inflammation and systemic injury in hepatic Kupffer cells [32,33,34]. Additionally, liver injury activates hepatic stellate cell (HSCs) proliferation, which enhance transforming growth factor β (TGF-β) secretion and collagen synthesis, thus forming extracellular matrix deposition and advanced fibrogenesis [35,36].

#### 2.2.4. Hepatic Metabolism Disorder

Numerous studies have revealed that alcohol consumption is correlated with iron overload and hepcidin synthesis downregulation in Kupffer cells and hepatocytes in the liver, while hepcidin was proposed as a key mediator in iron homeostasis [37,38]. Moreover, alcohol can abolish the protective effect of hepcidin in situations of iron overload by rendering hepatic hepcidin synthesis insensitive to total body iron levels [38]. Excessive iron can act synergistically with alcohol to induce the oxidative stress and lipid peroxidation, increasing transferrin receptor 1 (TfR1) expression to promote intestinal iron absorption; thus, the additive effect of iron absorption and deposition could potentiate progressive liver damage [26,39].

### 2.3. Genetic Factors

The genetic impact on alcohol use disorder (AUD) and ALD development was elucidated in previous studies, as individual variation exists after chronic alcohol consumption [40]. Several genome-wide association studies (GWAS) have identified several genetic risk loci for ALD development, including Patatin-like phospholipase domain-containing-3 (PNPLA3) gene, which is the major risk factor for the progression of ALD, and to a lesser extent, transmembrane 6 superfamily member 2 (TM6SF2) and membrane-bound O-acyltransferase domain-containing protein 7 (MBOAT7), are the key determinants of ALD progression [41,42,43]. Meanwhile, a splice variant in hydroxysteroid 17-β dehydrogenase 13 (HSD17B13) was identified as a candidate for protection against ALD in a recent study [44].

#### 2.3.1. PNPLA3

PNPLA3 is predominantly expressed in adipose tissue and is closely related to lipid metabolism, regulation of energy homeostasis, and the maintenance of membrane integrity [45]. It is also highly synthesized in HSCs and is responsible for retinyl ester hydrolysis [46]. The rs738409 variant (C.444 C > G p.Ile148Met) in the PNPLA3 gene results in hydrolytic function reduction, fat accumulation, and further liver inflammation injury [47]. This variant is more frequent in the Hispanic population and particularly sensitive to fatty liver diseases, although it has also been investigated in NAFLD and HCC [47,48]. A previous meta-analysis provided evidence for a significant role for rs738409 in PNPLA3 in ALD progression [49]. In addition, the presence of rs738409 in PNPLA3 is associated with an increased risk of HCC development in patients with cirrhosis due to ALD, with an estimated risk of twofold after adjusting for other confounders (e.g., age, sex, body mass index) [50,51].

#### 2.3.2. TM6SF2

TM6SF2 is mainly expressed in the liver and intestine and is presumed to be involved in lipid turnover and metabolism [52]. The rs58542926 (c.499C > T p.Glu167Lys) genetic variant is associated with reduced triglyceride-rich lipoprotein secretion and increased liver triglyceride concentration, which has been investigated as a determinant of NAFLD or advanced liver fibrosis development in previous studies [53,54,55]. Notably, TM6SF2 rs58542926 combined with PNPLA3 rs738409 may have additive value in predicting ALD cirrhosis progression [56]; additionally, both of them had a potential risk for HCC development in ALD patients [51,57]. It is postulated that lipid accumulation, followed by lipid peroxidation, inflammatory cell response, oxidative stress, and DNA damage are also important steps in HCC development [58].

#### 2.3.3. MBOAT7

MBOAT7 is an enzyme that transfers fatty acids between phospholipids and lysophospholipids and is involved in the phospholipid acyl-chain remodeling pathway [59]. The rs641738 C > T variant in the MBOAT7-TMC4 locus is associated with phosphatidylinositol acetylation disturbance, further hepatic inflammation, and fibrosis progression, and has been identified as a risk factor for NAFLD or non-alcoholic steatohepatitis (NASH) formation [60,61,62]. Similarly, MBOAT7 combined with TM6SF2 and PNPLA3 gene variants were identified as risk factors in ALD cirrhosis development in a GWAS study [43], although inconsistent findings were revealed in a recent study, which may need more evidence for validation [63].

#### 2.3.4. HSD17B13

HSD17B13 encodes hepatic lipid droplet protein and plays a role in lipid metabolism. A recent in vitro study indicated that HSD17B13 may regulate HSCs activity and participate in liver fibrosis development [64]. The rs72613567 T > A variant results in shortened protein production and reduced enzymatic activity, which is associated with both ALD and NAFLD progression to cirrhosis or HCC, and decreased liver enzyme levels [44]. These findings were validated in two recent large cohort studies in different ethnic groups with alcohol use, suggesting the therapeutic potential of HSD17B13 in the future [65,66].

### 2.4. Epigenetic Modifiers

Epigenetics refers to a process that changes the gene activity without DNA sequence alteration, such as DNA methylation, histone modification, or RNA silencing by microRNAs (miRNAs) [67,68]. miRNAs are single-stranded, 19–22 nucleotide non-coding sequences that bind to the complementary sequence of messenger RNA molecules, regulating their function by inhibiting or silencing translation [69]. As shown in Table 1, the dysregulation of miRNAs correlates with ALD severity and prognosis via regulation of multiple functions, including intestinal permeability change, liver steatosis and fibrosis, and oxidative stress [8,16,70]. Other epigenetic mechanisms include acetylation and methylation of DNA mediated by ethanol [71]. The ongoing metabolism of ethanol produces excessive ROS and depletes glutathione, diverting the reaction from the production of methionine and S-adenosylmethionine (SAM), which is the predominant methyl donor in DNA methylation [72,73]. The hypomethylation of DNA further facilitates hepatocyte proliferation and tumorigenesis [74]. In addition, alcohol-exposed hepatocytes show decreased NAD^+^ levels, which is also an important co-factor in histone acetylation [73]. Decreased histone acetylation further impairs the Sirtuin 1 (SIRT1)–AMPK pathway, a hepatic lipid metabolism pathway, and results in fatty liver and advanced fibrosis formation [75]. Collectively, epigenetic modifiers may influence the ALD therapy response and can help in treatment plan modification, which needs to be confirmed in future studies [69].

### 2.5. Environmental Factors

Numerous epidemiological factors affect ALD development and progression [12]. Women are more vulnerable to ethanol-related liver damage than men after the same amount of alcohol consumption, possibly due to their lower ADH activity and higher body fat composition; moreover, the estrogen-mediated inflammatory response increases the risk of ethanol-related liver damage [8,113]. Obesity is the most widely recognized environmental risk factor in ALD and has a close interaction and additive effect with alcohol [114,115]. Obesity can affect the ethanol lipid solubility and adipose tissue pro-inflammatory cytokine production, leading to alcoholic steatohepatitis, whereas alcoholic fatty liver induces insulin resistance and promotes obesity [8]. Meanwhile, multiple components of metabolic syndrome, including waist circumference, smoking, and alcohol use are the risk factors for severe liver disease in a recent population-based study [116]. Other known comorbidities, including viral hepatitis, hereditary hemochromatosis, and HIV coinfection in patients with concomitant alcohol use have a higher risk of accelerated liver fibrosis and increased mortality of liver-specific disease [10,117,118,119]. Notably, caffeine intake may protect against ALD cirrhosis in recent studies [120,121]; additionally, drinking two cups of coffee per day was estimated to decrease half the risk of ALD cirrhosis in a meta-analysis [122]. 

### 2.6. ALD Spectrum

#### 2.6.1. Alcoholic Fatty Liver or Steatosis

The diagnosis of alcoholic fatty liver (AFL) disease is established in a patient with known AUD with hepatic steatosis seen on ultrasound combined with liver enzyme elevation and the absence of other causes of liver disease [12]. AFL development is regulated by several direct or indirect regulatory mechanisms, including PPARα and AMPK expression inhibition, and ACC activity enhancement, which results in increased fatty acid synthesis and deposition [22]. Fat vacuoles or macrovesicles can be observed in liver tissues under a microscope, which resolves rapidly after complete abstinence [123]. AFL are seldom diagnosed because of their asymptomatic or nonspecific symptoms [2].

#### 2.6.2. Steatohepatitis due to ALD

Steatohepatitis due to ALD is presumed to be a progressive liver lesion, which has an increased risk of cirrhosis and HCC [2]. The common histological features of steatohepatitis due to ALD include steatosis, ballooned liver cells containing large Mallory-Denk bodies, sclerosing hyaline necrosis, and lobular inflammation predominated by neutrophils, which are rarely seen in NAFLD [19,115]. The inflammatory environment enables further leukocyte infiltration, ROS formation, and hepatocyte injury. As the injury continues, the release of damage-associated molecular patterns (DAMPs) activates multiple immune reactions and promotes liver fibrosis or malignancy [9,124]. Similar to AFL, mild steatohepatitis rarely presents with clinical symptoms and can only be diagnosed by liver biopsy; further, the development of novel non-invasive tests is urgent for this condition [12].

#### 2.6.3. Alcoholic Hepatitis

Alcoholic hepatitis (AH) is a clinical entity associated with severe steatohepatitis due to ALD and has a high short-term mortality risk [125]. In addition to steatohepatitis, the histological features of AH may also include megamitochondria, satellitosis, and cholestasis, which are related to the prognosis [126]. Both adaptive and innate immune dysfunctions are more prominent in patients with AH than in those with non-alcoholic liver disease, contributing to a higher risk of neutrophilia, liver dysfunction, and multi-organ failure [127]. The clinical symptoms of AH are characterized by the presence of jaundice with/without other hepatic decompensation events (e.g., ascites, hepatic encephalopathy) in patients with ongoing alcohol use [12]. It is noteworthy that despite complete abstinence, a significant proportion of patients had persistent AH and even progressed to cirrhosis [128,129].

#### 2.6.4. Fibrosis/Cirrhosis due to Alcohol-Related Liver Disease

As the vicious cycle continues (i.e., liver injury and regeneration) in ALD patients with ongoing alcohol use, the acetaldehyde-protein adducts inactivate DNA repair, damage hepatocyte mitochondria, impair oxygen utilization, and further stimulate collagen band synthesis and deposition between central veins and portal areas, resulting in liver fibrosis [9,11]. Cirrhosis is further characterized by marked hepatic architectural distortion due to extensive fibrosis and regenerative nodule formation [2]. The clinical manifestations of the patients with fibrosis or cirrhosis due to ALD widely range from asymptomatic to various decompensation events (e.g., variceal bleeding, bacterial infection, and hepatorenal syndrome) [130]. Importantly, active excessive alcohol consumption was identified as the second most frequent triggering factor of acute-on-chronic liver failure (ACLF) in patients with chronic liver disease, including ALD [131,132].

## 3. Diagnosis

There is no unique presentation of ALD that can be completely distinguished from other etiologies of liver disease [133]. The European Association for the Study of the Liver (EASL) proposed that the diagnosis of ALD is suspected when the amount of regular alcohol consumption is >30 g/day in men or >20 g/day in women, combined with the presence of clinical and/or biological evidence suggestive of liver injury [2]. ALD should also be considered in patients with AUD presenting with extrahepatic manifestations, including symmetric peripheral neuropathy, pancreatitis, or cardiomyopathy [134]. For the purpose of early ALD detection because of their favorable prognosis, asymptomatic patients with heavy alcohol consumption are recommended for regular ALD screening transfer [135].

A liver biopsy is considered the gold standard to establish a definite ALD diagnosis, to assess the stage and prognosis of liver disease, and to exclude alternative causes of liver injury, as approximately 20% of the patients with chronic alcohol consumption and abnormal liver enzymes were proven to have other coexisting liver disease etiologies [125,136,137]. Nevertheless, the performance of liver biopsy may induce morbidities, including intrahepatic bleeding and pneumothorax in approximately 2% of patients and is generally not recommended in routine clinical practice in all patients with suspected ALD [136,138].

### 3.1. Non-Invasive Diagnostic Tools

Ultrasonography is a non-invasive, inexpensive, and widely available tool for early ALD screening, with a sensitivity of 60–94% and a specificity of 88–95% for detecting steatosis, although it varies significantly with the degree of fatty content [139,140]. Other limitations of ultrasound include its operator-dependent [140] and difficulty in differentiating fibrosis from steatosis [136]. Magnetic resonance imaging-based methods (e.g., magnetic resonance spectroscopy) are reliable methods for measuring hepatic fat with reproducibility; however, the cost and long examination time limit their use in routine examinations [2,141]. The controlled attenuation parameter (CAP) is a novel ultrasound-based elastography method used to measure hepatic steatosis [142]. CAP revealed modest discriminative ability in differentiating the steatosis severity in a recent ALD study, although further validation studies are warranted [143].

The measurement of liver stiffness by transient elastography (TE) has become a popular non-invasive method for screening liver fibrosis/cirrhosis in recent years [144]. The value of liver stiffness measured in ALD patients correlates well with the degree of fibrosis, portal pressure, and its complications [145,146]. Regardless of ethnicity, the implementation of TE was validated as a cost-effective screening tool and correlated with the prognosis in patients with different stages of liver disease [147]. Notably, several pathophysiological conditions may exist and interfere with the interpretation of TE values, such as hepatic neuroinflammation, congestion, and cholestasis [148,149,150]. Compared with the patients with other etiologies of cirrhosis, the patients with cirrhosis due to ALD had significantly higher liver stiffness values, presumably with a higher degree of fibrosis, although the serum aspartate aminotransferase (AST) and bilirubin levels may also be considered for value adjustment [151,152]. Recently, various elastographic methods, such as acoustic radiation force impulse (ARFI) and shear-wave elastography (SWE), have been developed to assess the degree of hepatic fibrosis [153]. Although limited ALD studies have compared these different elastographic methods, they may show a similar performance based on current evidence [136].

### 3.2. Blood Tests and Biomarkers

Various serum biomarkers have been proposed for the detection of chronic alcohol use in recent years, including indirect markers, such as alcohol-induced metabolic products (e.g., carbohydrate-deficient transferrin (CDT), 5-hydroxytryptophol (5-HTOL)), and direct markers, such as alcohol metabolites (e.g., ethyl glucuronide (EtG), ethyl sulfate (EtS), phosphatidylethanol (Peth), and fatty acid ethyl esters (FAEEs)) [154]. CDT has been the most commonly used biomarker to confirm chronic alcohol consumption for years, although the sensitivity and specificity vary considerably between different studies, and numerous confounders could influence the results, including age, sex, and the stage of liver disease [155,156]. EtG can be detected in the urine or hair, has a much longer detection window, and a higher specificity than CDT, although biological variability (e.g., medications and foods) still exists and may complicate the interpretation of the results [157,158]. Several studies have compared the detection ability of these biomarkers and revealed conflicting results [154,157,159,160]. It is proposed that the combination of these biomarkers, such as EtS in urine, FAEEs in hair, and PEth in serum, may improve the overall sensitivity and specificity and provide more reliable results [154]. Recently, the differential methylation of DNA in specific genes revealed its potential utility as a diagnostic marker of active alcohol consumption, and may provide another choice in molecule-based studies in ALD [161,162].

Conventional liver function tests (i.e., AST, alanine aminotransferase (ALT), γ-glutamyl transferase) are widely used for liver disease screening; additionally, an AST/ALT ratio greater than 1.5 is traditionally considered as a diagnostic biomarker in ALD [136,163]. However, this frequent lack of ethanol specificity or distinguishing of the disease pathology, while advanced fibrosis due to ALD may present with normal liver function tests [164]. In order to exclude alternative causes of liver injury, EASL recommended a series of blood examinations, including hepatitis B and C virus serology, autoimmune markers, transferrin, and α1-antitrypsin in the workup of ALD patients [2,125,155]. In patients with advanced fibrosis or cirrhosis due to ALD, serum albumin levels, coagulation function profiles, bilirubin levels, and white blood cell counts should also be collected to determine the severity of the liver injury [165]. 

As a distinct clinical syndrome widely ranges from a few signs or symptoms to liver failure, the need for novel diagnostic biomarkers in AH is urgent, including cytokines and microRNA [166]. Mallory-Denk bodies, the hallmark of alcoholic hepatitis and steatohepatitis, contain cytokeratin-18 (CK-18) and cytokeratin-19 (CK-19), while the serum CK-18 and CK-19 levels were increased in AH patients in previous studies [167]. Meanwhile, the caspase-cleaved CK18 fragment M30 and M65 had modest predictive ability in biopsy-proven AH patients [168]. Interleukin-22 (IL-22) is a member of the IL-10 cytokine family and plays a protective role against liver injury in AH by anti-apoptosis, anti-oxidation, anti-fibrosis, and liver regeneration promoting effects [169,170]. The potential role of IL-22 as a diagnostic biomarker of AH has been investigated in several current studies [171]. Circulating miRNAs are another emerging biomarker candidate, as they regulate the inflammatory response and Kupffer cell activity (e.g., miR-155), hepatocyte damage (e.g., miR-122), cell proliferation and apoptosis (e.g., miR-30a) [82,172,173]. The majority of these miRNAs are packaged into exosomes or extracellular vesicles in the circulation; therefore, the lack of standardized extracellular vesicle isolation and sample collection/handling methods is currently a major obstacle [163]. 

The development of biomarkers for excess connective tissue deposition activity and progression of fibrosis due to ALD is another important issue [155]. Type I and type III collagens are the principal collagen deposited in liver tissues in response to alcohol injury; additionally, their derivatives of procollagen (e.g., type III procollagen aminopropeptide (PIIINP), type I procollagen aminopropeptide (PINP), and type I procollagen carboxypropeptide (PICP)) have been investigated as the biomarkers of fibrosis progression in ALD patients [35]. Furthermore, hyaluronic acid (HA) synthesized by HSCs and matrix metalloproteinases (MMPs) together with tissue inhibitor of metalloproteinases (TIMPs) also play a key role in fibrogenesis and fibrolysis, extracellular matrix formation, and degradation [174]. Recently, the enhanced liver fibrosis (ELF) test combined with HA, PIIINP, and TIMP-1, which revealed a similar diagnostic accuracy to the histological examinations in fibrosis progression evaluation in ALD patients [175]. The ELF also demonstrated superior fibrosis discrimination ability compared with different biological tests, such as the AST to platelet ratio index (APRI) or fibrosis-4 (FIB-4) index [176,177]. In summary, a combination of non-invasive diagnostic tests with serum-based fibrosis biomarkers will likely emerge as the mainstay diagnostic tool in the future [136].

## 4. Management

### 4.1. General Consideration

Complete alcohol abstinence is the cornerstone and improves the clinical outcomes in the treatment of all ALD stages [2,12]. Multidisciplinary management with the use of pharmacological therapy and behavioral intervention, as well as lifestyle modification, is recommended for prolonged abstinence attainment [178,179]. Sudden discontinuation or reduction in alcohol consumption may lead to alcohol withdrawal syndrome (AWS) in alcohol-dependent patients within 24 h after the last drink [180]. AWS includes varying degrees of autonomic hyperactivity symptoms and may progress to life-threatening conditions such as seizures, coma, cardiac arrest, and even death [2,180]. Benzodiazepines are considered as the standard treatment for AWS, and other drugs, such as β-blocker or α2-agonists can be used as adjunctive therapy [180]. Regular physical exercise and nutritional assessments should be incorporated into the abstinence strategy [115]. Meanwhile, public health policies to reduce the general population exposed to alcohol, such as a price-based taxation, increasing the legal age for buying alcohol, alcohol availability limitations, and advertising restrictions, have shown their effectiveness [181].

Malnutrition is a common complication in ALD patients, defined as the loss of body weight, muscle or fat mass, muscle strength, visceral protein levels, and immune function [182]. Malnutrition was estimated to be present in 50% of outpatient ALD patients and nearly all hospitalized ALD patients, and is associated with poorer prognosis [183,184]. The reason for ALD patients with malnutrition is often multifactorial, including an altered olfactory and gustatory perception, appetite-related hormone alterations, and gut microbiota changes [184]. The nutritional support in hospitalized ALD patients mainly focuses on the increased protein/calorie intake via various routes (e.g., enteral or parenteral), replacement of amino acids, and micronutrients [182,183]. Patients with ALD should receive proper nutritional status assessment and nutritional support when malnutrition is recognized [12]. In addition, efforts should be made to ensure adequate outpatient follow-up [184]. 

### 4.2. Pharmacology Therapy in Relapse Prevention

The current medications for relapse prevention in ALD patients are highlighted in Table 2. Among these, only disulfiram, naltrexone, and acamprosate were both approved by the US and Europe [26,133]. Disulfiram inhibits the enzyme acetaldehyde dehydrogenase, producing high levels of acetaldehyde following alcohol consumption [185]. Naltrexone is an opioid receptor antagonist that can reduce dopamine release in the reward system [186]. Both disulfiram and naltrexone undergo hepatic metabolism; hence, they should be avoided in patients with advanced ALD because of the fear of hepatotoxicity [133]. Acamprosate is the calcium salt of N-acetyl homotaurine, which is mainly metabolized through kidney excretion [186]. Acamprosate was proposed as a treatment option for patients with AUD, although it failed to demonstrate the treatment efficacy in a recent network meta-analysis [186]. Nalmefene, another opioid receptor antagonist approved only in Europe, could be considered in patients with early stages of ALD, where abstinence is not feasible in these patients [187,188]. Baclofen, a gamma-aminobutyric acid-B (GABA-B) agonist, increases the abstinence rate and prevents relapse in patients with AUD [189]. Notably, baclofen is the only AUD pharmacotherapy tested in patients with established cirrhosis, although several studies have reported inconsistent results [190,191,192,193]. Nevertheless, a recent international consensus statement recommended the consideration use of baclofen to treat AUD in patients with advanced ALD [194].

### 4.3. Specific Treatment for Alcoholic Hepatitis

#### 4.3.1. Corticosteroids

Corticosteroids are the most widely studied interventions in severe AH; they can change the cytokine balance, reduce pro-inflammatory cytokines, and increase anti-inflammatory cytokines [211]. The Maddrey discriminant function is the mostly widely used scoring system for AH, and a cut-off value of 32 identifies patients for initiating corticosteroid therapy [12,212]. Subsequently, the Lille score, a prognostic model calculated by baseline data and a change of serum bilirubin on day 7 of corticosteroid therapy, can be used to assess the response to corticosteroid [12,213]. A Lille score (ranges from 0 to 1) ≥ 0.45 indicates non-response to corticosteroid, and patients should discontinue therapy and consider other treatment strategies [12]. Numerous randomized trials regarding corticosteroid efficacy in AH have demonstrated conflicting results, likely due to the heterogeneity of the studied group and lack of power to detect survival differences [214]. A landmark multicenter randomized trial revealed that corticosteroids confer only modest 28-day survival benefits but not long-term outcomes, which was confirmed in a recent meta-analysis [215,216]. Infection is the most worrisome complication of corticosteroids, ranging approximately 20% in AH patients receiving corticosteroid treatment, and may offset its therapeutic benefit [217,218]. Therefore, the use of corticosteroids is restricted to the AH patients without infection, which eliminates a substantial proportion of patients [126].

#### 4.3.2. Antioxidants

Since oxidative stress plays a central role in the hepatotoxicity and pathogenesis of ALD and AH, antioxidants are of theoretical interest in AH treatment [219]. N-acetylcysteine (NAC) restores the glutathione stores and limits oxidative stress, although no additional survival benefit was observed in AH patients treated with NAC alone [220,221]. Notably, the combination of NAC and prednisolone compared with prednisolone alone revealed an improvement in the one-month survival, as well as a decreased incidence of hepatorenal syndrome and infections [222]. Metadoxine is another promising antioxidant that can aid in glutathione metabolism and inhibit hepatic steatosis [223]. The combination of metadoxine with steroids or pentoxifylline showed a significant improvement in both short-and long-term survival, and prolonged abstinence maintenance was observed [224,225]. Future large studies are required to validate these findings and provide further suggestions [126]. 

#### 4.3.3. Liver Regeneration

The counterbalance of cell death in AH is the liver regeneration capacity, which is supported by bone marrow-derived stem cells and hepatic progenitor cells [226,227]. Granulocyte-colony stimulating factor (G-CSF) can stimulate the bone marrow to produce and release stem cells into the circulation and facilitate liver progenitor cell proliferation [228]. Several small clinical studies have demonstrated that G-CSF administration is associated with increased survival rates and a decreased risk of infection in AH patients [229,230]. Furthermore, a recent Indian randomized trial showed the mortality benefit of G-CSF compared with a placebo in patients with steroid-nonresponsive AH [231]. Nevertheless, this favorable effect was not duplicated in the currently available US or European studies, which requires more evidence in future trials [232,233]. Obeticholic acid, the farnesoid X receptor (FXR) agonist, regulates bile acid homeostasis, decreases cholestasis, and further modulates liver regeneration, although the clinical trial was terminated due to its hepatotoxicity [228,234]. 

#### 4.3.4. Anti-Inflammatory/Anti-Apoptosis

The highly inflammatory condition of AH involves crosstalk between various signaling pathways, including pro-inflammatory cytokine and chemokine production, such as TNF-α, IL-1, and IL-6 [126]. Pentoxifylline (PTX), a non-selective phosphodiesterase inhibitor, can decrease TNF-α and other pro-inflammatory cytokine production and was initially tested as a therapeutic agent in AH [235]. However, growing evidence has demonstrated that the use of PTX is associated with a reduction in the hepatorenal syndrome development, but not the mortality benefit [215,216]. Based on current evidence, the use of PTX in AH treatment was no longer recommended in the current guidelines [2,12,133]. Similarly, studies of TNF-α inhibitors, such as infliximab and etanercept, were terminated early due to infection-related mortality [236,237]. Other novel therapeutic agents, including IL-1β antagonists, IL-22 agonists, and C-C chemokine antagonists, have been investigated in various clinical trials [238,239].

One of the alcohol-induced hepatocyte injury pathways is the process of apoptosis and macrophage activation mediated by endoplasmic reticulum stress, the mitochondrial pathway, and the caspase-dependent pathway, which further triggers abnormal liver tissue repair and inflammation [24,240]. Emricasan is a pan-caspase inhibitor that has been studied in the animal models of liver injury [241]. A recent multicenter randomized trial showed an improved three-month survival after treatment with emricasan compared with a placebo in patients with cirrhosis, regardless of the etiologies [242]. Nevertheless, a dose-ranging study of emricasan may be needed in further AH trials, as patients with severe AH have altered hepatic metabolism and pharmacodynamics [243]. Selonsertib is an oral apoptosis signal regulating kinase-1 (ASK-1) enzyme inhibitor, which can theoretically attenuate apoptosis and cytokine signaling [238]. Selonsertib failed to show survival benefit or liver function improvement compared with steroids in a recent study of patients with severe AH [244].

#### 4.3.5. Gut-liver Axis Targeting

Alcohol-induced gut-liver axis dysfunction was initiated with an intestinal microbiome composition change and permeability increase, which stimulates bacterial translocation, LPS release, and endotoxemia [245]. An animal study demonstrated that the susceptibility to ALD can be manipulated by intestinal microbiome implantation in AH patients, further confirming the role of microbiota in AH pathogenesis [246]. The strategy to reverse these processes may be achieved by intestinal decontamination [238]. IMM-124E is a purified hyperimmune bovine colostrum enriched with IgG antibodies against LPS [247]. It can modulate immune cell function, thus alleviating liver injury in experimental models; additionally, the clinical trials of AH are ongoing [241]. The administration of probiotic or antibiotic therapy is another potential approach for AH management [241]. A pilot study in the patients with mild AH demonstrated that the administration of *Bifidobacterium bifidum* and *Lactobacillus plantarum 8PA3* for five days significantly reduced liver biochemistry profiles and restored gut flora [248]. Other trials on probiotic or antibiotic, including amoxicillin clavulanate, *Lactobacillus rhamnosus*, and rifaximin, are under investigation [17]. Another promising therapeutic option is fecal microbiota transplantation (FMT). It revealed increased survival, reduced pathogen levels, and increased levels of beneficial bacterial strains in steroid-resistant patients with severe AH [249]. In summary, microbiome modulation has emerged as a novel and practical therapeutic approach for AH treatment [126,228,250].

### 4.4. Other Novel Therapies

#### 4.4.1. MicroRNA Modulation

As mentioned earlier, miRNAs are packaged into exosomes or extracellular vesicles and are expressed as the regulators of target proteins involved in a variety of oxidative stress, inflammatory responses, and lipid metabolism during ALD development [8]. The most widely studied miRNAs in ALD are miR-122 and miR-155 [251]. In mature hepatocytes, miR-122 constitutes 70% of all miRNAs, and notably, it possibly has pleiotropic roles in ALD pathogenesis, as it could sensitize monocytes to LPS stimulation and increases the pro-inflammatory cytokine levels in ethanol-treated hepatocytes [88]. Moreover, it protects hepatocytes from ethanol-induced damage by reducing hypoxia inducible factor 1 α (HIF-1α) levels [90]. Miravirsen, an miR-122 inhibitor, was previously investigated in hepatitis C treatment and may also have therapeutic potential in ALD [252,253]. Another important miRNA is miR-155, a major regulator of increased Kupffer cell activation and TNF-α production, and is also involved in ethanol-induced liver fibrosis and steatohepatitis by mediating the peroxisome proliferator-activated receptor response element (PPRE) and PPARα pathway [97]. The inhibition of miR-155 can lead to decreased ethanol-induced sensitivity of Kupffer cells to LPS in vivo [98]. Currently, there are no clinical trials regarding miRNA targeting in ALD treatment. Also, their roles as a main or adjunct therapeutic regimen also need more evidence for validation in the future [251].

#### 4.4.2. Mesenchymal Stem Cell

Stem cell transplantation therapy is another potential therapeutic option for ALD, especially in liver fibrosis [9]. Mesenchymal stem cells (MSCs) can provide support to hematopoietic stem cells and initiate the hematopoiesis process, and they also play an important role in organ homeostasis in past studies [254]. Regarding liver regeneration treatment, the benefits of MSCs intervention in ALD include parenchymal cell trans-differentiation and hepatocyte proliferation, promotion of regeneration ability, modulation of inflammatory responses, and inhibition of liver fibrosis [18]. Compared with miRNAs, a variety of clinical studies on MSCs therapy in different etiologies of liver disease have been conducted in recent years, including the ALD spectrum [255]. A phase 2 pilot study used bone marrow-derived MSCs for treating patients with cirrhosis due to ALD, which revealed a significant histological and quantitative improvement of hepatic fibrosis at 12 weeks after MSCs injection [256]. Another multicenter study used bone marrow-derived MSCs for the treatment of cirrhosis due to ALD; it showed significant improvement in histologic fibrosis and liver function after a longer follow-up period [257]. Taken together, the implementation of MSCs can be an attractive strategy in ALD treatment if their survival rate and activity could be further enhanced in the future field of regenerative medicine [258].

### 4.5. Liver Transplantation

Patients with end-stage ALD who respond poorly to medical therapies may be considered for liver transplantation (LT) [259]. A prior prospective multicenter study demonstrated that early LT improved the six-month survival probability in patients with severe alcoholic hepatitis, nonresponsive to standard corticosteroid therapy [260]. Notably, ALD is the leading indication of LT, accounting for 15–20% of all LTs in the US and Europe [261,262]. Although the survival rates of LT in ALD patients were poor in the 1980s, they have become comparable to those in patients transplanted for other indications [262]. It is noteworthy that long-term alcohol consumption often damages other organs and presents with extra-hepatic manifestations (e.g., cardiomyopathy, chronic kidney disease, pancreatitis, sarcopenia, and peripheral neuropathy), which should be evaluated before surgery as they may negatively impact post-transplantation outcomes [261]. In addition, complete abstinence is required before surgery, as it allows time for the liver to recover from alcohol-related toxic effects; also, the patient’s commitment to sobriety can be assessed [211]. Nevertheless, there is no consensus regarding the duration of abstinence in LT guidelines [2,12,133,261].

The major obstacles to LT in ALD patients include the scarcity of donors, immunologic rejection, complexity and costs of surgery, and most importantly, the ethical issue [263]. ALD is widely considered a self-inflicted disease in the general public, even practicing physicians, and the allocation of organs is more likely to prioritize patients with acquired diseases that are less directly related to behavior [2,263]. The concerns of relapse after LT is another consideration affecting their willingness to provide LT for ALD patients, as LT cures the liver disease but not the underlying AUD [264]. It was estimated that approximately 20–25% of ALD patients relapsed within five years of LT [265]. A multidisciplinary approach, including a relapse prevention program, may help reduce the risk of recidivism [266].

## 5. Conclusions

ALD is a major cause of liver disease worldwide, causing an extensive public health burden related to health, social, and economic harm. Growing evidence supports that ALD development is not solely explained by excessive alcohol consumption, but also comprises complex factor interactions, including genetic, epigenetic, and environmental modifier effects. Most early phase patients are asymptomatic, and the combination of non-invasive diagnostic tools and serum-based biomarkers has the potential to improve their prompt diagnosis. Complete abstinence and nutritional support remained the mainstay of ALD treatment, and a multidisciplinary implementation including psychosocial support and pharmaceutical intervention is necessary, which can help to prevent alcohol relapse. Moreover, the application of public health policies is a practical method for reducing the general population ALD risk. The miRNAs, mesenchymal stem cells, and other emerging novel therapies have potential roles in advanced-stage ALD treatment. Future translational science-based research and clinical trials are urgently required to identify the biomarkers for liver regeneration assessment, inflammation evaluation, and organ failure prediction in ALD patients [10].

## Figures and Tables

**Table 1:** Current experimental models of miRNAs involved in ALD pathogenesis.

miRNA	Sample Source	ALD Severity	Dysregulation	Function
miR17-92 cluster [76]	Human HSC cell line Rat model	Cirrhosis and fibrosis due to ALD	Various (e.g., miR-17a decreased, miR-92 increased)	HSC activation. Inhibited MeCP2/TGFβRII expression
miR-21 [77,78,79]	Mice and rat model Human HSC and hepatocyte cell line Human HCC cell line	Alcoholic liver injury HCC	Increased	HSC activation, hepatocyte survival, transformation, and remodeling Increased α-SMA, FASLG, DR5 expression
miR-26a [80]	Mice model, Human HCC cell line	Alcoholic liver injury HCC	Decreased	Promote cytoprotective Autophagy, Target Beclin-1, DUSP4, DUSP5
miR-27a [81,82]	Human blood, Human monocyte	Alcoholic liver injury Alcoholic hepatitis	Increased	M2 Monocyte polarization, Downregulate sprouty2, Increase CD206
miR-29 [83]	Human blood, Murine model	Cirrhosis due to ALD CCl4-induced hepatic fibrogenesis	Decreased	Downregulate HSC and collagen expression Modulate intestinal permeability
miR-34a [84,85,86,87]	Human hepatocyte and cholangiocyte, Mice model	Fibrosis due to ALD HCC	Increased	Hepatocyte steatosis, inflammation and fibrosis Decrease caspase 2 and SIRT1
miR-122 [88,89,90]	Human blood, Mice model	Steatohepatitis, fibrosis, or cirrhosis due to ALD HCC	Decreased	Downregulate HIF-1α, cyclinG1, Bcl-w, Reprogram monocyte to LPS stimulation, Regulate lipid metabolism
miR-125b [85,91]	Human HCC cell line and tissues, Rat model	HCC, Alcoholic liver injury	Decreased	Decrease PIGF expression, Distort MMP-2, MMP-9 expression
miR-126 [92,93]	Human HCC tissues Rat model	HCC, CCl4-induced hepatic fibrogenesis	Decreased	Downregulate HSC activity, Regulate VEGF-A, PI3K, p-AKT, cyclin D1 activity
miR-155 [94,95,96,97,98]	Mice model, Murine hepatocytes and Kupffer cells	Steatohepatitis, fibrosis, or cirrhosis due to ALD	Increased	Kupffer cells activation Induce TNF-α and NF κB activity, Mediate PPAR-α pathway, Induce C/EBPβ activity
miR-181b-3p [99]	Mice model, Rat model Murine Kupffer cells	Alcoholic liver injury	Decreased	Sensitize Kupffer cells to TLR4-mediated cytokine production, Modulate importin α5 expression
miR-182 [100,101]	Human liver tissues Mice model	Alcoholic hepatitis, Alcoholic liver injury	Increased	Promote hepatocyte inflammation, Upregulate CCL20, CXCL1, IL-8, Cyclin D1
miR-199 [102,103]	Human HCC tissues Rat liver sinusoidal endothelial cells	HCC, Alcoholic liver injury	Decreased	Regulate hepatocyte inflammation and immune cells infiltration, Attenuate HIF-1α and ET-1 expression
miR-200a [103]	Mice hepatocyte cell line, Mice model	Alcoholic liver injury	Increased	Modulate hepatocyte apoptosis, Decrease ZEB2 expression
miR-212 [104,105]	Human gut epithelial cells, Mice model	Advanced ALD Alcoholic liver injury	Increased	Disrupt tight junctions integrity, gut leakage Downregulate ZO-1 expression
miR-214 [106]	Human HCC cell line Rat hepatocyte, Rat model	HCC Alcoholic liver injury	Increased	Induce hepatocyte oxidative stress, Repress GSR and POR activity
miR-217 [107,108]	Murine macrophage Mice model	Alcoholic liver injury	Increased	Hepatocyte steatosis and inflammation, Downregulate SIRT1
miR-223 [109]	Human blood, Mice model	Chronic alcohol use, Alcoholic liver injury	Decreased	Limit neutrophil infiltration and ROS production Inhibits IL-6–p47phox–ROS pathway
miR-291b [110]	Rat model, Human monocyte	Alcoholic liver injury Alcoholic hepatitis	Increased	Sensitize monocyte to TLR4 signaling, Downregulate Tollip expression
miR-378 [111]	Mice model, Human HCC tissues	CCl4-induced hepatic Fibrogenesis, HCC	Decreased	Suppress HSC activation Decrease Gli3 expression
miR-497 [112]	Mice model, Mice hepatocyte	Alcoholic liver injury	Decreased	Alleviate bile acid synthesis, Reduce Btg2, Yy1 levels

**Table 2:** Relapse prevention medications in patients with ALD.

Medication	Dose	Mechanisms	Adverse Effects	Comment
Disulfiram [186,195]	250–500 mg/day	Inhibit acetaldehyde dehydrogenase	Hepatotoxicity, metallic taste, polyneuritis, skin allergy	Effective treatment, No studies in advanced ALD
Naltrexone [187,196]	50 mg/day	Opioid receptor antagonist	Hepatotoxicity, headache, nervousness, abdominal cramps, myalgia	Effective treatment, No studies in advanced ALD
Acamprosate [187,197]	1998 mg/day	NMDA receptor antagonist Glutamatergic receptor modulator	Diarrhea, insomnia, anorexia, asthenia	Avoid in severe renal impairment, No studies in advanced ALD
Nalmefene [189,198]	10–20 mg/day	Opioid receptor antagonist	Nausea, vomiting, dizziness	Reduction of heavy drinking
Baclofen [191,192,193,194,199,200]	15–60 mg/day	GABA-B receptor agonist	Drowsiness, fatigue, headache, dry mouth	Off-label use, Consider in advanced ALD, Low-dosage preferred
Topiramate [201,202,203]	75–200 mg/day	GABA receptor agonist, glutamate receptor antagonist	Drowsiness, dizziness, loss of coordination, anorexia	Reduction of heavy drinking, No studies in ALD
Gabapentin [204,205,206]	600–1800 mg/day	Inhibit presynaptic calcium channel Influence GABA and glutamate activity	Dizziness, fatigue, ataxia, drowsiness, diplopia	A recent RCT showed good efficacy, Consider as second-line medication
Ondansetron [207]	4–8 µg/kg/day	5-HT3 receptor antagonist	Constipation, headache, drowsiness	No recommendations in guidelines
Sertraline [208]	50–200 mg/day	SSRI	Anorexia, dry mouth, dyspepsia, insomnia	May be helpful in selective patient group
Sodium oxybate [209]	50–100 mg/kg/day	GABA receptor agonist	Dizziness, sedation, asthenia	Approved in Italy and Austria, Risk of abuse
Varenicline [210]	0.5–2 mg/day	Partial nAChR agonist	Nausea, vomiting, insomnia, headache	May be effective in smokers with AUD

## Abbreviations

5-HTOL	5-hydroxytryptophol
α-SMA	α smooth muscle actin
ACC	adiponectin and acetyl-CoA carboxylase
ACLF	acute-on-chronic liver failure
ADH	alcohol dehydrogenase
AFL	alcoholic fatty liver
AFRI	acoustic radiation force impulse
AH	alcoholic hepatitis
ALD	alcohol-related liver disease
ALDH	aldehyde dehydrogenase
ALT	alanine aminotransferase
AMPK	AMP-activated protein kinase
APRI	aspartate aminotransferase to platelet ratio index
ASK-1	apoptosis signal regulating kinase-1
AST	aspartate aminotransferase
AUD	alcohol use disorder
AWS	alcohol withdrawal syndrome
Btg2	b-cell translocation gene 2
CAP	controlled attenuation parameter
CCl4	carbon tetrachloride
CCL20	C-C motif chemokine ligand 20
CDT	carbohydrate deficient transferrin
C/EBPβ	CCAAT/enhancer-binding protein β
CK-18	cytokeratin-18
CK-19	cytokeratin-19
CXCL1	C-X-C motif chemokine ligand 1
CYP2E1	cytochrome P450 2E1
DAMPs	damage-associated molecular patterns
DR5	death receptor 5
DUSP	dual specificity phosphatase
EASL	European association for the study of the liver
Egr-1	early growth response protein 1
ELF	enhanced liver fibrosis
ET-1	endothelin-1
EtG	ethyl glucuronide
EtS	ethyl sulfate
FAEEs	fatty acid ethyl esters
FASLG	fas ligand
FIB-4	fibrosis-4
FMT	fecal microbiota transplantation
FXR	farnesoid X receptor
GABA-B	gamma-aminobutyric acid-B
G-CSF	granulocyte-colony stimulating factor
GSR	glutathione reductase
GWAS	genome-wide association studies
HA	hyaluronic acid
HCC	hepatocellular carcinoma
HIF-1α	hypoxia inducible factor 1α
HIV	human immunodeficiency virus
HSCs	hepatic stellate cells
HSD17B13	hydroxysteroid 17-β dehydrogenase 13
IL	interleukin
LPS	lipopolysaccharide
LT	liver transplantation
MBOAT7	membrane-bound O-acyltransferase domain-containing protein 7
MeCP2	methyl-CPG binding protein 2
MEOS	microsomal ethanol-oxidizing system
miRNA	microRNA
MMP	matrix metalloproteinases
MSCs	mesenchymal stem cells
NAC	N-acetylcysteine
nAChR	nicotinic acetylcholine receptors
NAD	nicotinamide adenine dinucleotide
NAFLD	nonalcoholic fatty liver disease
NASH	nonalcoholic steatohepatitis
NF-κB	nuclear factor kappa-light-chain-enhancer of activated B cells
NMDA	N-methyl-d-aspartate
Nrf-2	nuclear factor erythroid 2-related factor 2
PICP	type I procollagen carboxypropeptide
PINP	type I procollagen aminopropeptide
PIIINP	type III procollagen aminopropeptide
PEth	phosphatidylethanol
PDGF	platelet-derived growth factor
Phox	phagocytic oxidase
PI3K	phosphoinositide 3-kinase
PIGF	placenta growth factor
PNPLA3	patatin-like phospholipase domain-containing-3
POR	cytochrome P450 oxidoreductase
PPARα	peroxisome proliferator activated receptor α
PTX	pentoxifylline
RCT	randomized controlled trial
ROS	reactive oxygen species
SAM	s-adenosylmethionine
SIRT1	sirtuin 1
STAT3	signal transducer and activator of transcription 3
SSRI	selective serotonin reuptake inhibitors
SWE	shear-wave elastography
TE	transient elastography
TfR1	transferrin receptor 1
TGF-β	transforming growth factor β
TGFβRII	type II transforming growth factor-β receptor
TIMPs	tissue inhibitor of metalloproteinases
TLRs	toll-like receptors
TM6SF2	transmembrane 6 superfamily member 2
TNF-α	tumor necrosis factor α
VEGF	vascular endothelial growth factor
WHO	world health organization
Yy1	yin yang 1
ZEB2	zinc finger E-box binding homeobox 2
ZO-1	zonula occludens 1
